# Colorectal cancer-derived microvesicles modulate differentiation of human monocytes to macrophages

**DOI:** 10.1186/s12967-016-0789-9

**Published:** 2016-02-02

**Authors:** Monika Baj-Krzyworzeka, Bożenna Mytar, Rafał Szatanek, Marcin Surmiak, Kazimierz Węglarczyk, Jarek Baran, Maciej Siedlar

**Affiliations:** Department of Clinical Immunology, Institute of Peadiatrics, Medical College, Jagiellonian University, 265 Wielicka str., 30-663 Cracow, Poland; Division of Molecular Biology and Clinical Genetics, Department of Internal Medicine, Medical College, Jagiellonian University, Cracow, Poland

**Keywords:** Tumour-derived microvesicles, Macrophages, Monocytes, Regulatory cells

## Abstract

**Background:**

Tumour-derived microvesicles (TMVs) are important players in tumour progression, modulating biological activity of immune cells e.g. lymphocytes, monocytes and macrophages. This phenomenon is particularly interesting in the progression of colon cancer, as macrophages in this type of tumour are relevant for the recovery processes. In the present study, the role of colon cancer cell-derived microvesicles in monocyte differentiation and activity profile (polarization) was investigated.

**Methods:**

Monocyte-derived macrophages (MDM) were differentiated in vitro in the presence of TMVs obtained from colon cancer: Caco-2, SW620, LoVo or SW480 cell lines and analysed according to their morphology and biological functions, as defined by cytokine secretion, reactive oxygen intermediate (ROI) production and cytotoxic activity against respective colon cancer cells.

**Results:**

Monocytes differentiated with TMVs exhibited morphological and phenotypical characteristics of macrophages. An early contact (beginning with the first day of the in vitro culture) of monocytes with TMVs resulted in increased IL-10 secretion and only slightly elevated TNF release. Early, or prolonged contact resulted in low ROI production and low cytotoxicity against tumour cells. On the other hand, late contact of MDM with TMVs, stimulated MDM to significant TNF and IL-12 secretion, ROI production and enhanced cytotoxicity against tumour cells in vitro. In addition, differences in MDM response to TMVs from different cell lines were observed (according to cytokine secretion, ROI production and cytotoxicity against tumour cells in vitro). Biological activity, STATs phosphorylation and microRNA profiling of MDMs indicated differences in their polarization/activation status which may suggest mixed polarization type M1/M2 with the predominance of proinflammatory cells after late contact with TMVs.

**Conclusions:**

Macrophage activity (polarization status) may be regulated by contact with not only tumour cells but also with TMVs. Their final polarization status depends on the contact time, and probably on the vesicle “cargo”, as signified by the distinct impact of TMVs which enabled the switching of MDM maturation to regulatory macrophages.

## Background

Colorectal cancer is the third most common cancer and the fourth most frequent cause of cancer deaths worldwide [1, 2]. This type of tumour occurs generally after the age of 50 and in most cases is sporadic, however, the occurrence of genetic or epigenetic causes along with the inflammatory microenvironment support colorectal cancer development [3]. The infiltrating leukocytes, mainly monocytes, which give rise to macrophages, are the hallmarks of this process [4]. The majority of macrophages infiltrating neoplastic tissue (TAM, tumour associated macrophages) have phenotype characteristics of M2 polarized cells, which are known to participate in each step of cancer progression including dissemination, seeding and metastasis formation [5]. The level of their infiltration is used as an independent prognostic factor in many tumour types [6]. However, it should be noted that in case of colorectal cancers, a strong macrophage infiltration does not necessarily correlate negatively with patients’ survival [4, 7, 8]. This may be associated with proinflammatory TAMs, which play an antitumour role, leading to a favourable prognosis [9]. Different biological properties of macrophages could be associated with distinct factors (growth factors, cytokines etc.) in their microenvironment during the differentiation process [10]. The tumour microenvironment is a very complex system of cell to cell interplay complemented by cellular interactions with the extracellular environment. Tumour cells are essential players in these processes, however, other factors should be also taken under consideration. One of the most intriguing factors are ubiquitous extracellular vesicles (EVs). During tumour progression, monocytes in blood, as well as macrophages in the tumour bed, are exposed to EVs released by nearby cells [11, 12] to body fluids [13, 14]. EVs are defined as membrane fragments of various shapes released by cells during their lifespan. Using the size and origin criteria, EVs are classified into two groups: exosomes, which are smaller (30–100 nm), more homogeneous in size and released by the endosomal compartment, and ectosomes, also known as microvesicles (MVs) [15], which are larger (0.1–1 μm) [16] and mainly originated from plasma membranes. EVs are also released by tumour cells, thus, impacting the activity of blood monocytes and TAM in tumour bed [17]. Based on their cargo (proteins, growth factors, mRNA and microRNAs) EV are regarded as “messengers” [12–15], which may affect biological activity of macrophages. miRNAs deserve special attention as they may regulate all differentiation steps and change the activation status of macrophages [18]. In the present study, we asked whether TMVs released by colon cancer cell lines (with different growth potential) may influence monocyte differentiation, thus, affecting their activity/polarization status. In another words, we asked, if TMVs, as tumour “go-between” present in body fluids/tumour environment, may direct/educate monocytes during their differentiation. Our results indicate an important role of TMVs in this process and suggest that TMVs origin and the time of their “first contact” with monocytes/macrophages are crucial for their ultimate activity and function.

## Methods

### Isolation of monocytes

Human peripheral blood mononuclear cells (PBMCs) were isolated from EDTA-blood of healthy donors by the Ficoll/Isopaque (Pharmacia, Uppsala, Sweden) density gradient centrifugation. Monocytes were separated from PBMCs by counter-flow centrifugal elutriation with the JE-6B elutriation system equipped with a 5 ml Sanderson separation chamber (Beckman-Coulter, Palo Alto, CA), as previously described [19]. Monocytes were suspended in RPMI 1640 culture medium (PAA Laboratories, Pasching, Germany) with gentamycin (Sigma, St. Louis, MO) (25 μg/ml). Purity of monocytes was over 95 %, as judged by staining with anti-CD14 mAb (BD Biosciences Pharmingen, San Diego, CA) and flow cytometry analysis (FACSCanto BD Biosciences Immunocytometry Systems, San Jose, CA). The study was approved by the local Jagiellonian University Ethical Committee (No. KBET/160/b/2011).

### Isolation of TMVs

TMVs were obtained from the following human colon cancer cell lines: Caco-2, SW480, SW620 and LoVo as previously described [20]. The cell lines were a generous gift from prof. Caroline Dive (Paterson Institute for Cancer Research, The University of Manchester). Cell lines differed in malignancy potential as Caco-2 was described as poorly aggressive, SW480-with low metastatic ability, SW620-high metastatic ability and LoVo-undifferentiated. Cells were cultured by bi-weekly passages in RPMI 1640 (Caco-2 and LoVo) or DMEM (SW480 and SW620) (PAA) with 5 % FBS (foetal bovine serum, Biowest, Nuaille, France) centrifuged before the use at 50,000×*g* for bovine-derived MVs depletion. Cell lines were regularly tested for *Mycoplasma* sp. contamination by using PCR-ELISA kit according to the manufacturer’s protocol (Roche, Mannheim, Germany). Supernatants from well-grown cell cultures were collected, centrifuged at 2000×*g* for 20 min to remove cell debris and then centrifuged again at 50,000×*g* (RC28S, Sorvall, Newton, CT) for 1 h at 4 °C. Pellets were washed twice in PBS to remove FBS and finally resuspended in serum-free medium. Quantification of TMVs proteins was evaluated by the Bradford method (BioRad, Hercules, CA). TMVs were tested for endotoxin contamination by the Limulus test according to the manufacturer’s instruction (Charles River Laboratories, Inc., Wilmington, MA) and stored at −20 °C until use. As an additional control for some experiments MVs from non-malignant urothelial cells HCV-29 were used. To simplify, TMVs were named according to their cell line origin e.g. TMVs released from Caco-2 as TMV_Caco2_, from LoVo-TMV_LoVo_, from SW480-TMV_SW480_, from SW620 as TMV_SW620_ and from HCV-29 as MV_HCV_.

### Differentiation of monocytes to monocyte-derived macrophages (MDM)

**Fig. 1:**
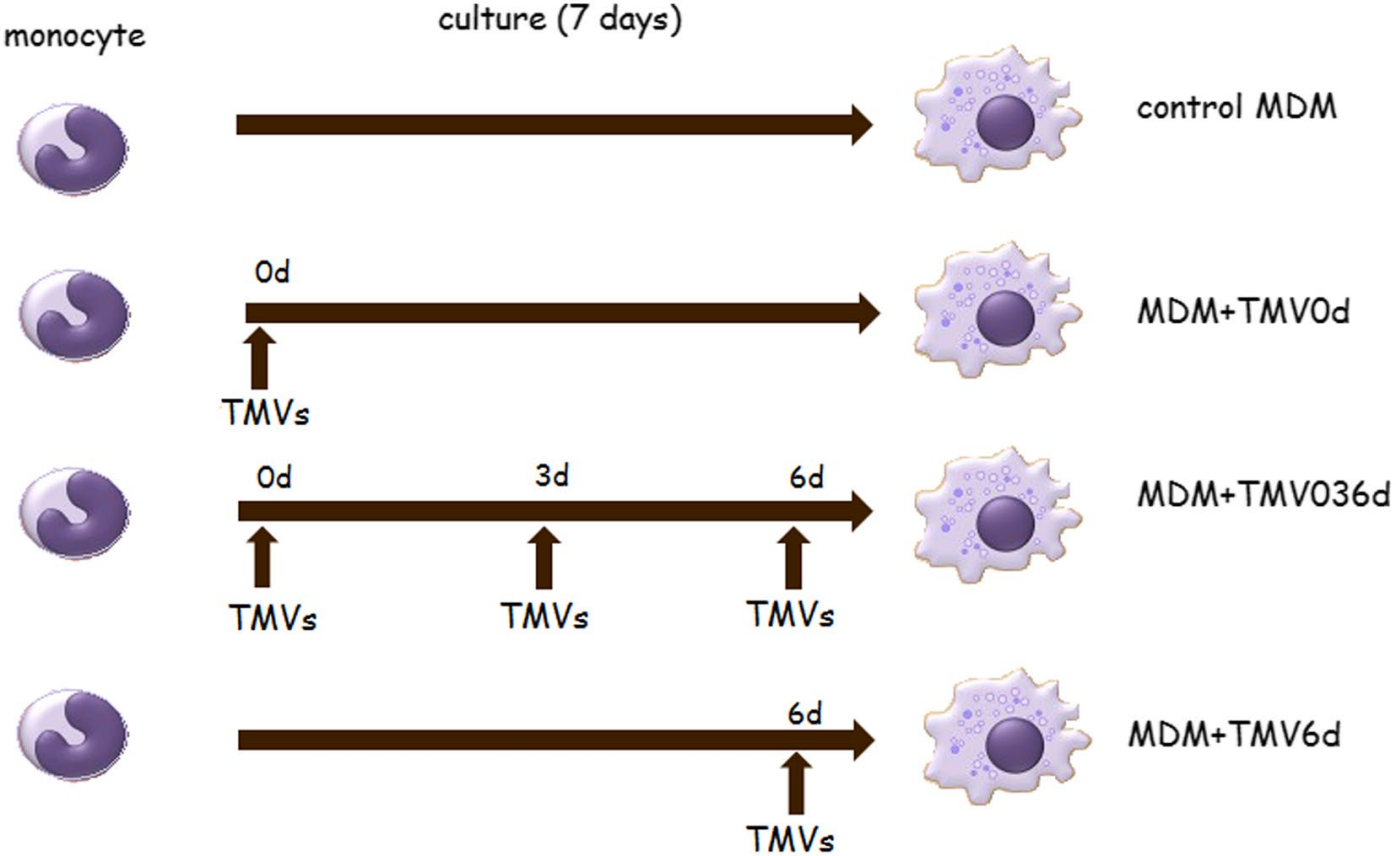
Scheme of experiments. Control MDM were obtained from human peripheral blood monocytes cultured for 7 days without growth factors. MDM + TMV0d were obtained from human peripheral blood monocytes supplemented at day 0 with appropriate TMVs. Similarly, MDM + TMV036d were exposed to TMVs at day 0, 3 and 6 and MDM + TMV6d only at day 6. All other culture conditions were the same

Blood monocytes were cultured for 7 days in 24-well low attachment plates (Corning Incorporated, Corning, NY) at the density of 1×10^6^/well at 37 °C, 5 % CO_2_ in humidified atmosphere. In the preliminary experiments, RPMI 1640 medium supplemented with FBS (low in endotoxin and MV-depleted) was established as adequate for differentiation of human monocytes to MDM. Also, the dose of TMVs (3 μg/ml) that had no impact on the viability of MDM (see below) was established during the preliminary experiments. TMVs (3 µg/ml) were added to the monocyte culture in three different combinations: (i) at day 0 (abbreviated as MDM + TMV0d), (ii) at days 0, 3, 6 (MDM + TMV036d), (iii) at day 6 (MDM + TMV6d) (Fig. 1). After 3 days of culture, half of the medium was removed and replaced with freshly prepared one. Our previous results indicated that TMVs supplemented at day 0 were completely engulfed after 24 h, so the risk to remove them was unlikely [20]. Monocytes cultured alone (without TMVs) were used as a control (equivalent to M0 macrophages) [21] and defined as control MDM. In parallel, MDM were differentiated in the presence of GM-CSF (1000 U/ml, Immunotools GmbH, Friesoythe, Germany) or M-CSF (10 ng/ml, Peprotech, Princeton, NY) which have been described to induce an M1 and M2 like phenotype, respectively [21]. As an additional control MV_HCV_ and fluorescent beads [0.4 μm Fluoresbrite Yellow Green (YG) carboxylate microspheres, Polysciences, Warrington, USA] were used, to check the impact of normal MVs and the engulfment process on MDM differentiation. After 7 day culture, cell viability was determined by Annexin V (BD Pharmingen) binding and TO-PRO-3 (Invitrogen, Waltham, MA) staining; only cells with viability above 95 % were used for further testing.

### Morphology and phenotype of MDM

The morphology of macrophages was investigated daily by phase contrast microscopy (600× magnification) Olympus IX70 (Olympus Corp. PA). MDM morphology was assessed using cytospin slides stained for 3 min with the Wright’s dye (Merck, Darmstadt, Germany) at the last day of culture by light microscopy (Olympus BX51). Phenotype analysis was performed at day 7 using flow cytometry. The following monoclonal antibodies (mAbs) were used: APC-labelled anti-CD14, FITC-labelled anti-CD33, -CD80, -CD15, PE-labelled anti-CD1a, -CD206, -CD86, -CD11b, PerCP-labelled anti-HLA-DR, PerCP-Cy5.5-labelled anti-CD163, all from BD Pharmingen. Antibodies from R&D System: FITC-labelled anti-CD115 (MCSF-R), PE-labelled anti-CD36. For intracellular staining (CD68 FITC) MDM were fixed with Cytofix and permeabilised with PermWash buffers, both from BD Pharmingen. CD68 staining was done using mAb from Dako Cytomation (Glostrup, Denmark). Additionally, kinetics of CD206 expression was examined on a daily basis using flow cytometry. All antibodies were used in saturating concentrations with appropriate isotype-matched controls. MDM were incubated with mAbs for 30 min at 4 °C, washed and then analysed in FACSCanto flow cytometer.

### Cytokine secretion

Supernatants obtained on day 7 of the MDM culture were collected and the concentration of TNF, IL-10 and IL-12 was measured by the following matched mAbs pairs for ELISA (BD Pharmingen): for TNF-MAb1 (capture) and MAb11 (detection), for IL-10-JES3-9d7 (capture) and JES-12G8 (detection) and for IL-12p40/p70-C8.3 (capture) and C8.6 (detection). Recombinant human cytokines (all from BD Pharmingen) were used as standards. Tests were performed according to the manufacturer’s protocol and results were obtained using the ELISA reader (BioTek Instruments, Vinooski, VT) at 492 vs 630 nm wavelength. Detection level for TNF was 20 pg/ml, and 10 pg/ml for IL-10, IL-12p40/p70.

### microRNA (miR) expression

MDM differentiated in the presence of TMVs (TMV_SW480_, TMV_SW620_, TMV_LoVo_ and TMV_Caco2_) were tested for miR expression profile. The total RNA was extracted from MDM, MDM + TMVs (after 7 days of culture) and TMVs alone with *mir*Vana™ miRNA Isolation Kit (Ambion, Life Technologies, Austin, TX) according to the manufacturer’s protocol. The first strand cDNA was obtained from the total RNA (400 ng) samples with Megaplex™ RT Primers (pool A and B, Applied Biosystems, Foster City, CA). TaqMan Array Micro RNA Card Set v3.0 were used to detect expression of human miRs in preliminary studies. Real-time PCR was performed using the 7900HT System (Applied Biosystems). For miRs, which major differences in expression were detected in preliminary experiments (miR-9,-21,-155,-378,-511) individual real-time PCRs (TaqMan Gene Expression Assays, Applied Biosystems) were performed. All experiments were performed three times and the PCR reactions were performed in triplicates using the 7300 Real-Time PCR System (Applied Biosystems). Reverse transcription was performed as described above (Megaplex RT primers pool A were used for miR-9,-21,-155,-511 and U6 as a control, Megaplex RT primers pool B were used for miR-378 and U6). The fluorescent signals generated during the informative log-linear phase were used to calculate the relative amount of miR. U6 was used as a control for each PCR run and the miR expression was calculated as a fold difference from that of control MDM normalized by U6 results (2^−ΔΔCT^).

### Determination of ROI production by flow cytometry

The intracellular production of ROI was measured by flow cytometry using oxidation-sensitive fluorescent probe hydroethidine (HE, Sigma). After 7 days, MDM expanded alone or in the presence of TMVs (added in the described regimen) were preincubated with 10 µM HE for 30 min and stimulated with phorbol 12-myristate 13-acetate (4 µM PMA, Sigma) for 10 min, after which cells were washed and analysed immediately with FACSCanto flow cytometer.

### Western blotting

To assess the master regulators in signalling processes that lead towards M1 (STAT1, STAT5) or M2 (STAT3, STAT6) differentiation Western blotting technique was employed. Monocytes were stimulated with TMVs for 30′, 2 h, 4 h and 7 h at day 0 or day 6. After stimulation cells were lysed in M-PER lysing buffer (Pierce, Rockford, IL) containing protease inhibitor cocktail (Roche). As a control unstimulated monocytes/MDM were used. The concentration of samples was measured using the Bradford kit (Bio-Rad) as per manufacturer’s instruction. 20 µg of isolated protein was mixed with NuPAGE LDS Sample Buffer (4×) (Life Technologies, Carlsbad, CA) and NuPAGE Sample Reducing Agent (10×) (Life Technologies). Samples were heated (70 °C, 10 min) and electrophoresed in 12 % polyacrylamide gel containing SDS. Next, electrophoresed samples were transferred onto the polyvinylidene fluoride membrane (Bio-Rad). Then, after blocking for 1 h at room temperature in Tris buffered saline (TBS) with 0.1 % Tween-20 (Sigma, St. Louis, MO) and 1 % bovine serum albumin (BSA, Sigma) the membranes were incubated overnight at 4 °C with rabbit polyclonal antibodies: anti-phopspho-STAT1 (Tyr701), anti-phospho-STAT3 (Tyr705), anti-phospho-STAT5 (Tyr694), anti-total STAT1, anti-total STAT3 and anti-total STAT5 (all antibodies were purchased from Cell Signaling, Danvers, MA) diluted 1:1000. After incubation, membranes were washed in TBS supplemented with BSA and Tween-20 and incubated for 1 h at room temperature with secondary goat anti-rabbit antibody (dilution 1:2500) conjugated with horseradish peroxidase (Cell Signaling). The protein bands were visualized with the SuperSignal West Pico Chemiluminescence Substrate kit (Pierce) according to the manufacturer’s protocol and analysed with KODAK GEL LOGIC 1500 Digital Imaging System (KODAK, Rochester, NY).

### Cytotoxicity assay

MDM cytotoxicity against tumour cells was tested, as described previously [21]. Briefly, after 7 day culture, MDM (5×10^4^/well) grown alone or with TMVs were cocultured with the appropriate tumour cells (autologous) (2×10^4^/well) for 48 h, after which the culture medium was removed and 100 μl of 1, 3-[4,5-dimetylthiazol-2-yl]-2,5-diphenyltetrazolium bromide (MTT, 2 mg/ml, Sigma) dye solution was added for 4 h. The experiment was repeated 5 times in triplicates. Formed formazan was extracted with isopropyl alcohol (Fluka Chemie AG, Buchs, Switzerland) containing 0,04 N HCl and its content was measured spectrophotometrically (absorbance at 570 and 630 nm). The percentage of cytotoxicity was calculated according to the formula described previously [22].

### Statistical analysis

Statistical analysis was performed by nonparametric Mann–Whitney test. Differences were considered significant at p < 0.05.

## Results

### Monocytes in the presence of TMVs differentiate to macrophages

**Fig. 2:**
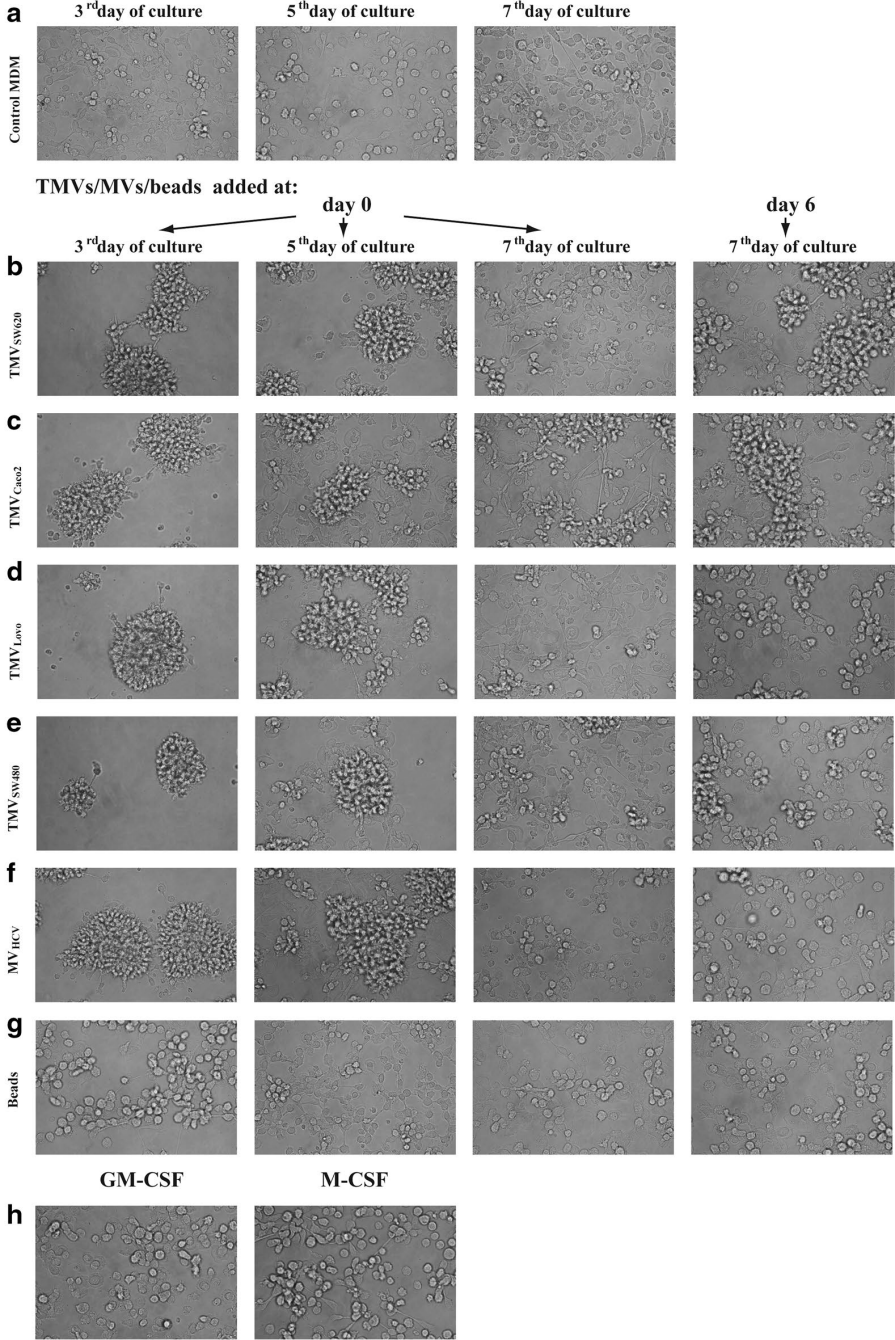
Morphology of MDM (at day 3, 5 and 7) differentiated in the presence of medium alone (**a**) or TMVs: TMV_SW620_ (**b**), TMV_Caco2_ (**c**), TMV_LoVo_ (**d**), TMV_SW480_ (**e**), MV_HCV_ (**f**), beads (**g**) and growth factors (**h**). One representative experiment out of ten is presented

The presence of TMVs during the culture significantly changes morphology of MDM (Fig. 2). It was observed that MDM form clusters after exposure to TMVs. When cultures were monitored daily, the “spreading out” of MDM from the clusters was observed at day 5 (Fig. 2). Control MDM as well as MDM after beads engulfment did not form clusters. Control MDM after 7 day culture exhibited heterogeneous shapes including elongated and round cells (Fig. 2a). MDM generated in the presence of TMV_SW620_ from day 0 (MDM + TMV_SW620_ 0d) were heterogeneous in shape (Fig. 2b). Accountable “fried egg”-shaped cells next to clusters were observed when TMV_SW620_ were supplemented at the final differentiation stage (MDM + TMV_SW620_ 6d, Fig. 2b). MDM + TMV_Caco2_ 0d were predominantly elongated (Fig. 2c). When TMV_Caco2_ were supplemented at day 6, MDM formed cluster-like structures (Fig. 2c). Similar pattern was observed for TMV_SW480_ (Fig. 2e), with “fried egg”-shaped cells more frequent at day 6. In the case of TMV_LoVo_, spindle-like cells were rare, while most of them resembled adherent macrophages (Fig. 2d), with “fried egg”-shaped morphology. In general, spindle-like MDM, which resembled cells cultured with M-CSF (Fig. 2h) were more frequent when TMVs were added at 0d. In contrast, MDM supplemented with TMV at day 6, looked similar to those differentiated with GM-CSF (Fig. 2h). MDM cultured with beads did not form cluster structures at all, and looked almost the same during the whole culture period (Fig. 2g). MDM supplemented with MV_HCV_ were similar despite the time of the contact with MV_HCV_ (Fig. 2f).

**Fig. 3:**
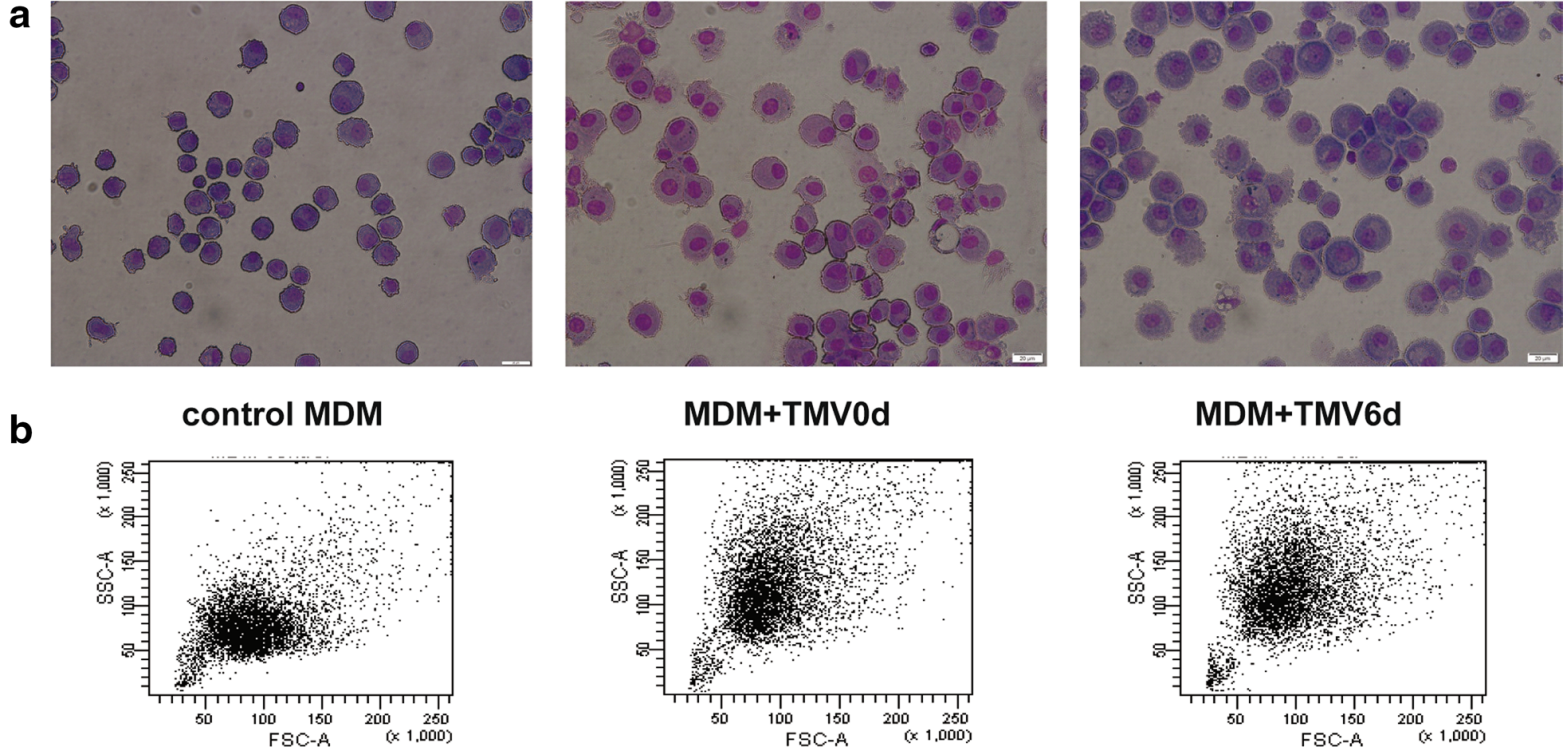
MDM morphology analysed by cytospin slides after the Wright’s staining (**a**) and flow cytometry FSC/SSC dot plot (**b**) of MDM differentiated in the presence of TMVs. *Left panel*-control, *middle panel*-MDM + TMV0d, *right panel*-MDM + TMV6d. (bar—50 µm). One representative example out of ten is presented

MDM differentiated in the presence of TMVs were usually bigger with higher granularity (as judged by flow cytometry and light microscopy) (Fig. 3a, b).

**Fig. 4:**
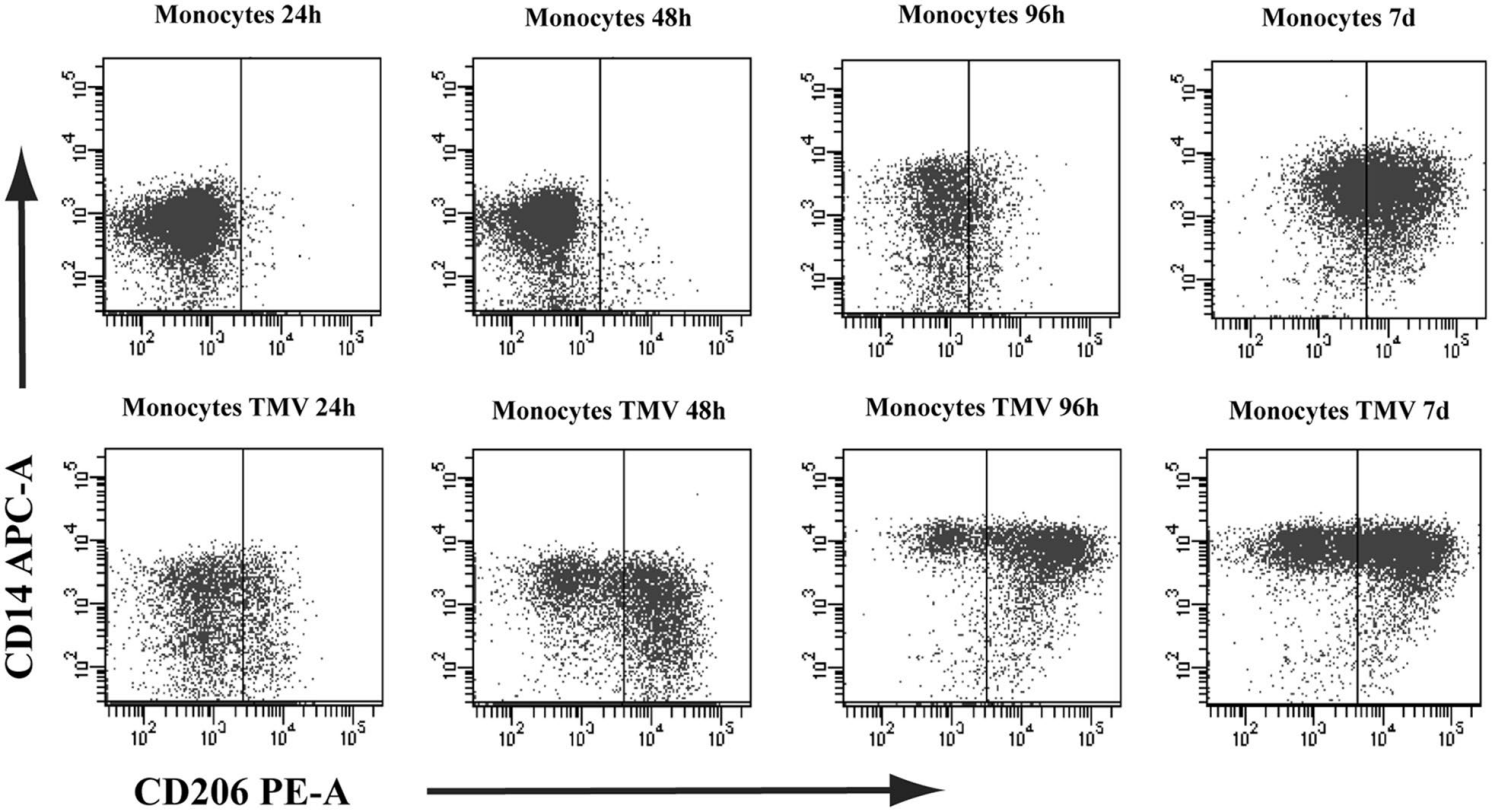
Expression of CD206 on MDM cultured alone (*upper panel*) or with TMV_LoVo_0d (*lower panel*) after 24, 48, 96 h and 7 days. One representative experiment out of 6 performed is presented. Similar kinetics of CD206 expression was observed for other TMVs

Control MDM as well as MDM + TMVs expressed CD14 molecules in the absence of the typical markers of dendritic cells (CD1a) and granulocytes (CD15) (data not shown). However, MFI of CD14 was significantly higher in MDM + TMV6d (28,117 ± 9906) in comparison to other groups (11,804 ± 6422 for control MDM and 12,096 ± 4991 for MDM + TMV0d). All MDM expressed macrophage-lineage marker CD68 (more than 90 %). Moreover, MDM were positive for CD11b and CD33 (70 and 33 %, respectively). Table 1 presents expression of selected markers on MDM cultured alone or with TMVs. Differences in marker expression were observed in the case of CD206, CD36, CD80, CD86, and CD163 after contact with particular TMVs and their significance was denoted with an asterisk (*p < 0.05, Table 1). Interestingly, we observed a dynamic increase in CD206 positive cells during the first days after contact with TMVs (Fig. 4). The annexin V binding (less than 3 % of cells), and the TO-PRO-3 staining (below 2 %) were low (not shown). As an additional control, MDM cultured with MV_HCV_ or fluorescent beads were used. No differences in the expression level of tested markers (as above) were observed between control MDM and MDM + MV_HCV_ or MDM + beads (data not shown).

**Table 1:** Phenotype analysis of MDM differentiated in the presence of TMVs. Percentages of positive cells (mean ± SD) in 5 independent cultures are presented

Marker	Control MDM	MDM + TMV0d				MDM + TMV036d				MDM + TMV6d			
TMVs from:		Caco-2	LoVo	SW620	SW480	Caco-2	LoVo	SW620	SW480	Caco-2	LoVo	SW620	SW480
HLADR	30 ± 18	46 ± 24	42 ± 20	38 ± 23	45 ± 23	45 ± 23	38 ± 14	29 ± 13	47 ± 20	15 ± 8	32 ± 16	15 ± 9	24 ± 17
CD80	3 ± 7	16 ± 17*	14 ± 14*	6 ± 7	41 ± 16*	14 ± 8*	24 ± 10*	10 ± 8	49 ± 18*	20 ± 19*	14 ± 9*	3 ± 3	26 ± 15*
CD86	56 ± 16	46 ± 9	51 ± 22	53 ± 19	27 ± 2*	43 ± 13*	54 ± 35	56 ± 19	18 ± 5*	81 ± 6*	72 ± 11*	67 ± 16	44 ± 17
CD36	79 ± 8	55 ± 7*	54 ± 11*	70 ± 10	63 ± 25	42 ± 9	46 ± 5*	66 ± 15	64 ± 17	71 ± 31	84 ± 8	81 ± 9	64 ± 25
CD163	15 ± 9	6 ± 1*	12 ± 4	6 ± 1*	3 ± 3*	6 ± 1*	11 ± 7	8 ± 3	4 ± 4*	24 ± 22	35 ± 12*	16 ± 16	6 ± 1*
CD206	48 ± 13	79 ± 16*	79 ± 12*	76 ± 21*	69 ± 6*	86 ± 6*	78 ± 11*	74 ± 25	73 ± 7*	62 ± 20	65 ± 17	65 ± 22	52 ± 7
CD115	10 ± 8	10 ± 7	11 ± 11	10 ± 7	17 ± 4	9 ± 4	9 ± 5	9 ± 4	9 ± 5	10 ± 9	6 ± 5	10 ± 9	3 ± 4

The statistical significance of differences between groups were calculated with Mann–Whitney test and denoted by an asterisk (* p < 0.05)

### MDM + TMV0d and MDM + TMV6d differ in microRNA expression

**Fig. 5:**
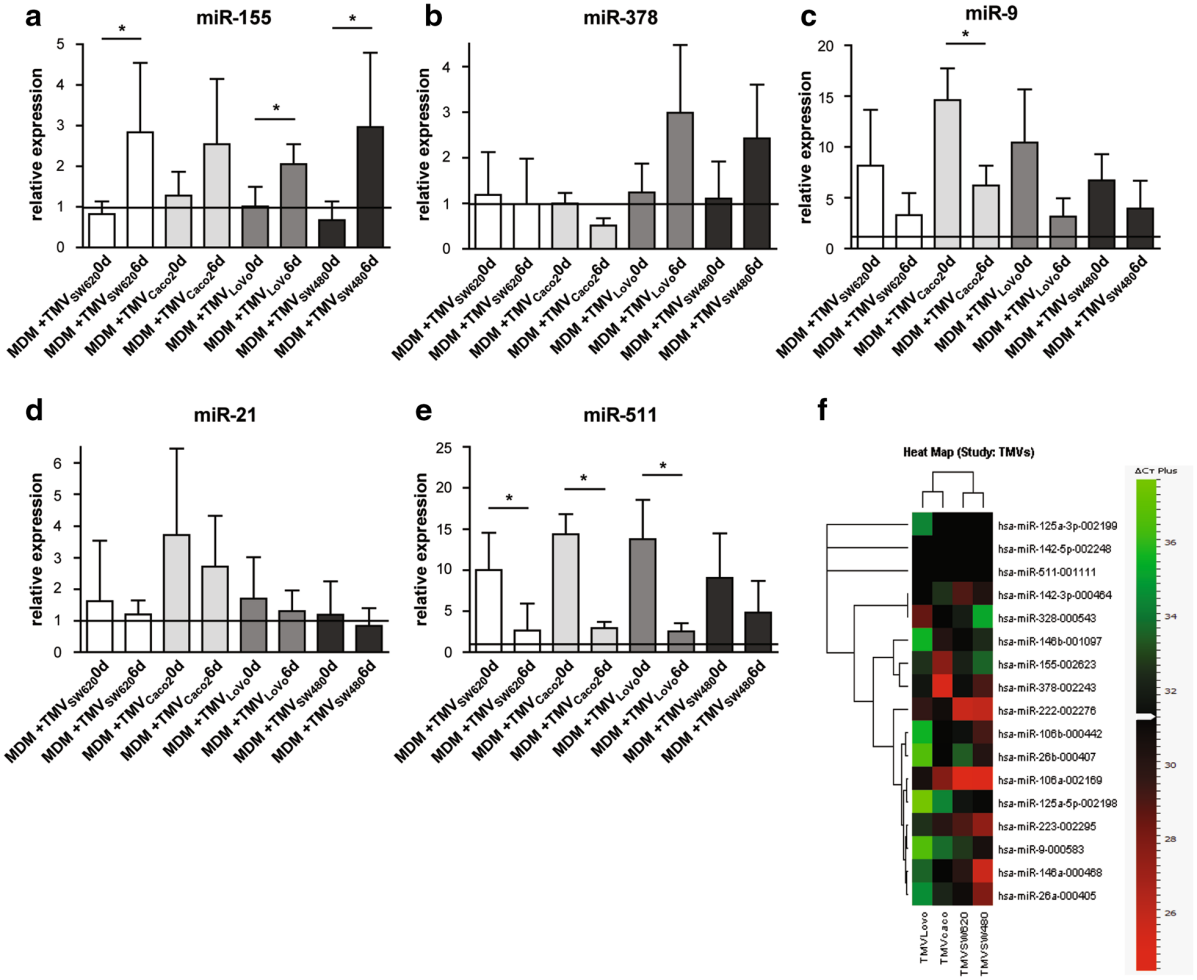
Expression of selected (involved in the MDM differentiation process) microRNAs in MDM + TMV0d and MDM + TMV6d vs control MDM (*black line* at level 1) presented as relative expression normalized to U6 (2^−ΔΔCT^): miR-155 (**a**), miR-378 (**b**), miR-9 (**c**), miR-21 (**d**), miR-511 (**e**). Heat map of microRNA involved in MDM differentiation process detected in TMVs alone (**f**). Data from 3 independent experiments, performed in triplicates (mean ± SD) are shown. *p < 0.05

Differences in expression of a number of miRs were observed including miR-9,-10a,-125a,-130a,-146a,-146b,-15,-155,-21,-222,-223,-27a,-328,-378,-511,-1254. The most important miRs for differentiation and polarization process were analysed in subsequent experiments with TaqMan probes. We confirmed that MDM + TMV6d expressed more miR-155 than MDM + TMV0d (Fig. 5a). Upregulation of miR-378 was detected only in MDM + TMV_LoVo_6d and MDM + TMV_SW480_6d in comparison to MDM + TMV0d (Fig. 5b). These two miRs are described to be associated with M1 polarization type. In parallel, we observed upregulation of miR-9 in all MDM + TMVs in comparison to control MDM, however expression of miR-9 in MDM + TMV0d was much higher than in MDM + TMV6d. Significant differences were observed in the presence of TMV_Caco2_ (Fig. 5c). A similar trend was observed in miR-21 expression (higher in MDM + TMV0d than in MDM + TMV6d, Fig. 5d). miR-9 and miR-21 expression may suggest M2-like polarization. Expression of miR-511 was significantly higher in MDM + TMV0d than in MDM + TMV6d which corroborates with the surface expression of CD206 (Fig. 5e).

### TMVs carry microRNA involved in macrophage differentiation

In parallel to miRs analysis in MDM, miRs expression in TMVs was determined, however, only miRs detected at CT below 31 were analysed (set arbitrarily, average CT presented in brackets). The TMV_Caco2_ carried miR: −106a (27, 92), −146b (30, 31), −155 (27, 82), −223 (30, 15), and −378 (25, 31), TMV_SW480_-miR-106a (24, 88), −106b (29, 29), −146a (25, 83), −21 (28, 16), −222 (26, 12), −223 (27, 62), −26a (28, 02), −378 (29, 23) and −9 (30, 77), TMV_SW620_ −106a (24, 59), −146a (30, 06), −21 (30, 28), −222 (25, 93), −223 (29, 13), TMV_LoVo_-miR-106a (30, 76), −222 (29, 74), −328 (28, 70). miR-511 was not detected. Data are presented as a heat map (Fig. 5f).

### TMVs induce secretion of cytokines by MDM

**Fig. 6:**
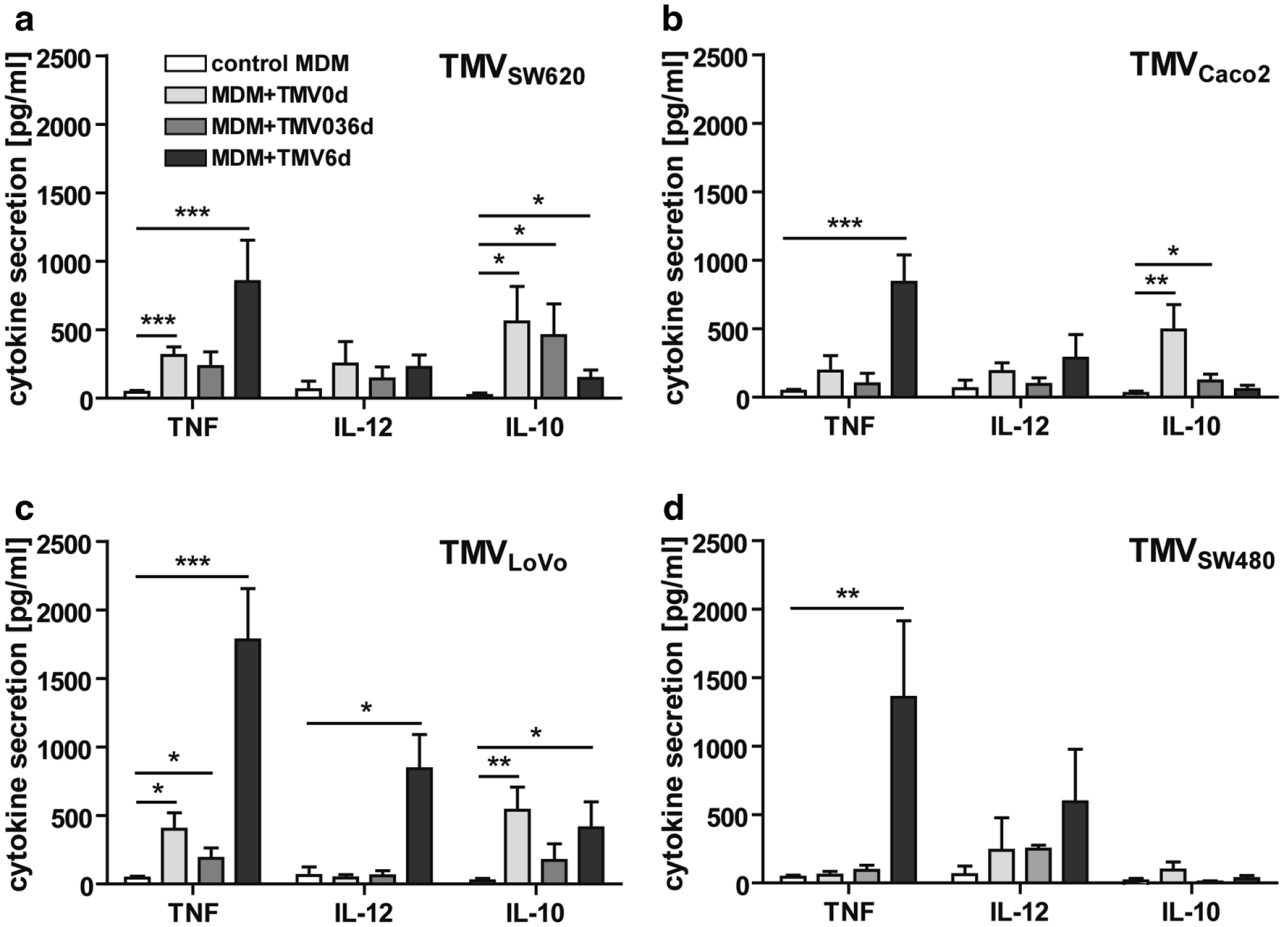
Secretion of cytokines (TNF, IL-12, IL-10) by MDM differentiated with TMVs. The supernatants were collected at day 7 and cytokines level was determined by ELISA method. Cytokine secretion by MDM culture with TMV_SW620_ (**a**), TMV_Caco2_ (**b**), TMV_LoVo_ (**c**), TMV_SW480_ (**d**) is presented. Data from 6 independent experiments (mean ± SD) are shown. *p < 0.05, **p < 0.001, ***p < 0.0001

Secretion of cytokines by MDM was tested after 7 days of culture with/without TMVs (Fig. 6). In general, MDM + TMV6d secreted significantly more TNF (850 ± 303, 839 ± 199, 1781 ± 74, 1355 ± 559 pg/ml for TMV_SW620_, TMV_Caco2_, TMV_LoVo_ and TMV_SW480_, respectively) than MDM + TMV0d (TMVs in order as above: 311 ± 62, 190 ± 114, 399 ± 119, 49 ± 24 pg/ml) and control MDM (43 ± 13 pg/ml). Also secretion of IL-12 by MDM + TMV6d was elevated, except for MDM + TMV_SW620_ (340 ± 199, 863 ± 280, 741 ± 457 pg/ml for TMV_Caco2_, TMV_LoVo_ and TMV_SW480_ respectively) in comparison to MDM + TMV0d (TMVs in order as above: 185 ± 68, 43 ± 24, 177 ± 151 pg/ml) and control MDM (26 ± 20 pg/ml). Interestingly, secretion of TNF and IL-12 was the lowest in MDM + TMV036d (except for TMV_SW480_). In the case of MDM + TMV0d, lower TNF secretion (see above) was accompanied by the increased secretion of IL-10 (558 ± 258, 491 ± 184, 564 ± 181, 96 ± 57 pg/ml for TMV_SW620_, TMV_Caco2_, TMV_LoVo_ and TMV_SW480_, respectively) whereas elevated TNF (see above) and lower IL-10 levels (162 ± 65, 54 ± 32, 476 ± 217, 33 ± 23 pg/ml, for TMV_SW620_, TMV_Caco2_, TMV_LoVo_ and TMV_SW480_, respectively) were observed in MDM + TMV6d. MV_HCV_ induced cytokines comparable with control MDM. Fluorescent beads did not induce TNF at all, while IL-10 and IL-12 secretion was similar to control MDM (data not shown).

### MDM + TMV6d produce high amounts of ROI

**Fig. 7:**
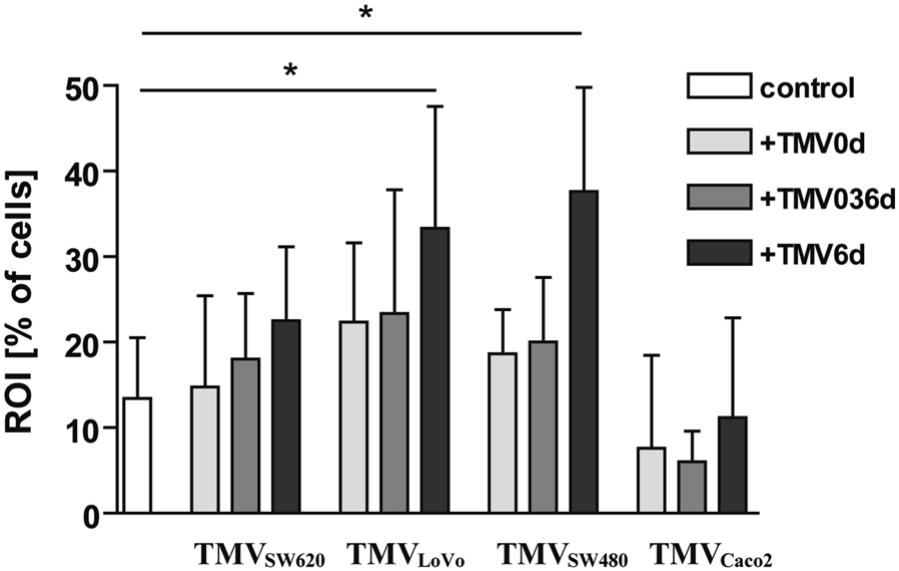
The intracellular production of ROI by MDM differentiated in the presence of TMVs. The level of ROI (mainly O_2_^−^) production was determined by flow cytometry. MDM were stimulated with PMA in the presence of HE for 30 min. Percentage of positive cells was presented (mean of six performed experiments). Data from 6 independent experiments (mean ± SD) are shown. *p < 0.05

MDM generated in the presence of TMVs produced ROI after PMA stimulation, however, the observed differences depended on the applied TMVs scheme. Significant increase of ROI production (mainly O_2_^−^) was observed only in MDM + TMV6d (22 + 9, 33 ± 14 and 37 ± 12 % of cells for TMV_SW620_, TMV_LoVo_ and TMV_SW480_ respectively) in comparison to the control MDM and MDM + TMV0d (13 ± 7). A similar profile of ROI production was observed in three of the four tested TMVs (TMV_SW480_, TMV_SW620_, and TMV_LoVo_). TMV_Caco2_ did not induce ROI production by MDM (Fig. 7).

### TMVs induce phosporylation of STAT1 and STAT3

**Fig. 8:**
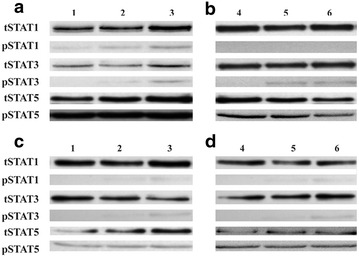
Western-blot analysis of STAT1, 3 and 5 phosphorylation. Monocytes isolated from blood of 2 donors were used: donor 1 (**a** and **c**), donor 2 (**b** and **d**). **a** and **b** represents MDM0d, **c** and **d** represents MDM6d. Control MDM (1 and 4), MDM + TMV_LoVo_ (2), MDM + TMV_SW480_ (3), MDM + TMV_SW620_ (5) MDM + TMV_Caco2_ (6). One representative experiment out of two performed is presented

To assess the polarization of MDM stimulated with TMVs, Western blot technique was employed to analyze STAT-1, 3, 5 and 6. Phosphorylation of STAT1, 3, 5 and 6 was tested during the time interval of 30′–7 h. A weak phosphorylation signal for STAT1 in MDM 0d and 6d stimulated with TMV_Lovo_ or TMV_SW480_ (Fig. 8a, c) for 7 h was detected. No phosphorylation of STAT1 was observed after TMV_Caco2_ and TMV_SW620_ (Fig. 8b, d) stimulation. STAT3 was phosphorylated on tyrosine (but not serine) after stimulation (7 h) with any type of TMVs regardless of the time of contact (MDM 0d, MDM6d). STAT5 was phosporylated quickly (30′ stimulation) in all samples (including control MDM), while phosphorylation of STAT 6 was not detected (not shown). Data are presented as a representative (out of two perfomed) Western blot (Fig. 8).

### TMVs change cytotoxic/cytostatic activity of MDM

**Fig. 9:**
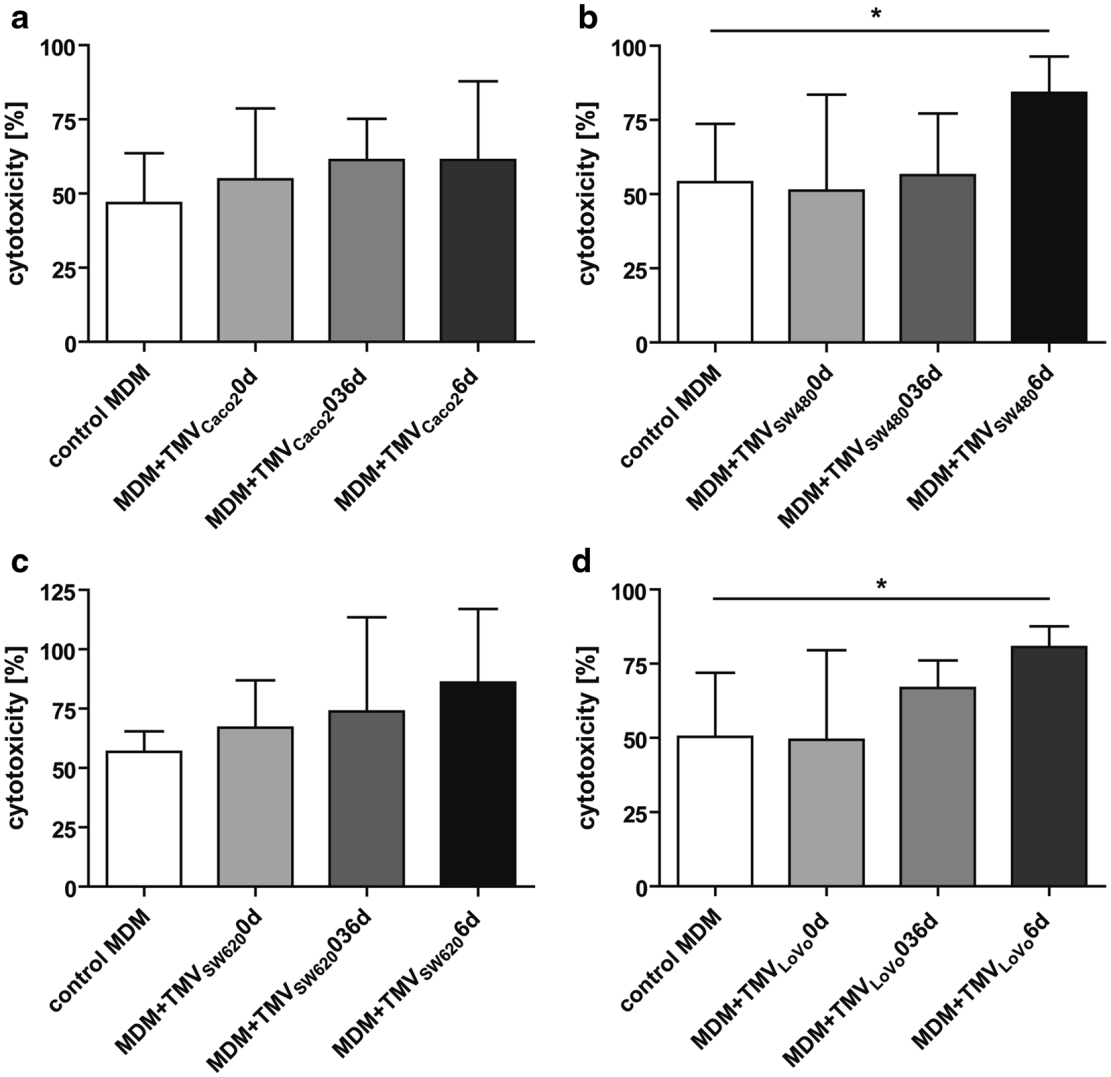
Cytotoxic/cytostatic activity of MDM against tumour cells. Monocytes were differentiated in the presence of TMVs and then cocultured with appropriate tumour cells for 48 h (**a** Caco-2,** b** SW480,** c** SW620,** d** LoVo). Proliferation of cells was determined by MTT reduction assay. The results of five independent experiments are shown (mean ± SD). *p < 0.05

Based on the obtained data, the cytotoxicity of control MDM in vitro against four tested colon cancer cell lines was app. 50 %. In general, MDM + TMV6d were more cytotoxic against tumour cells (86 ± 31, 61 ± 26, 80 ± 17, 84 ± 12 % of cells for TMV_SW620_, TMV_Caco2_, TMV_LoVo_, TMV_SW480_, respectively) than MDM + TMV0d (67 ± 19, 55 ± 23, 49 ± 30, 51 ± 32 % of cells for TMV_SW620_, TMV_Caco2_, TMV_LoVo_, TMV_SW480_, respectively) and control MDM (56 ± 8, 46 ± 16, 50 ± 21, 54 ± 19 % of cells for SW620, Caco-2, LoVo and SW480 cells, respectively). We did not observe significant changes in cytotoxicity of MDM + TMV0d or MDM + TMV036d (Fig. 9) in comparison to control MDM.

## Discussion

**Fig. 10:**
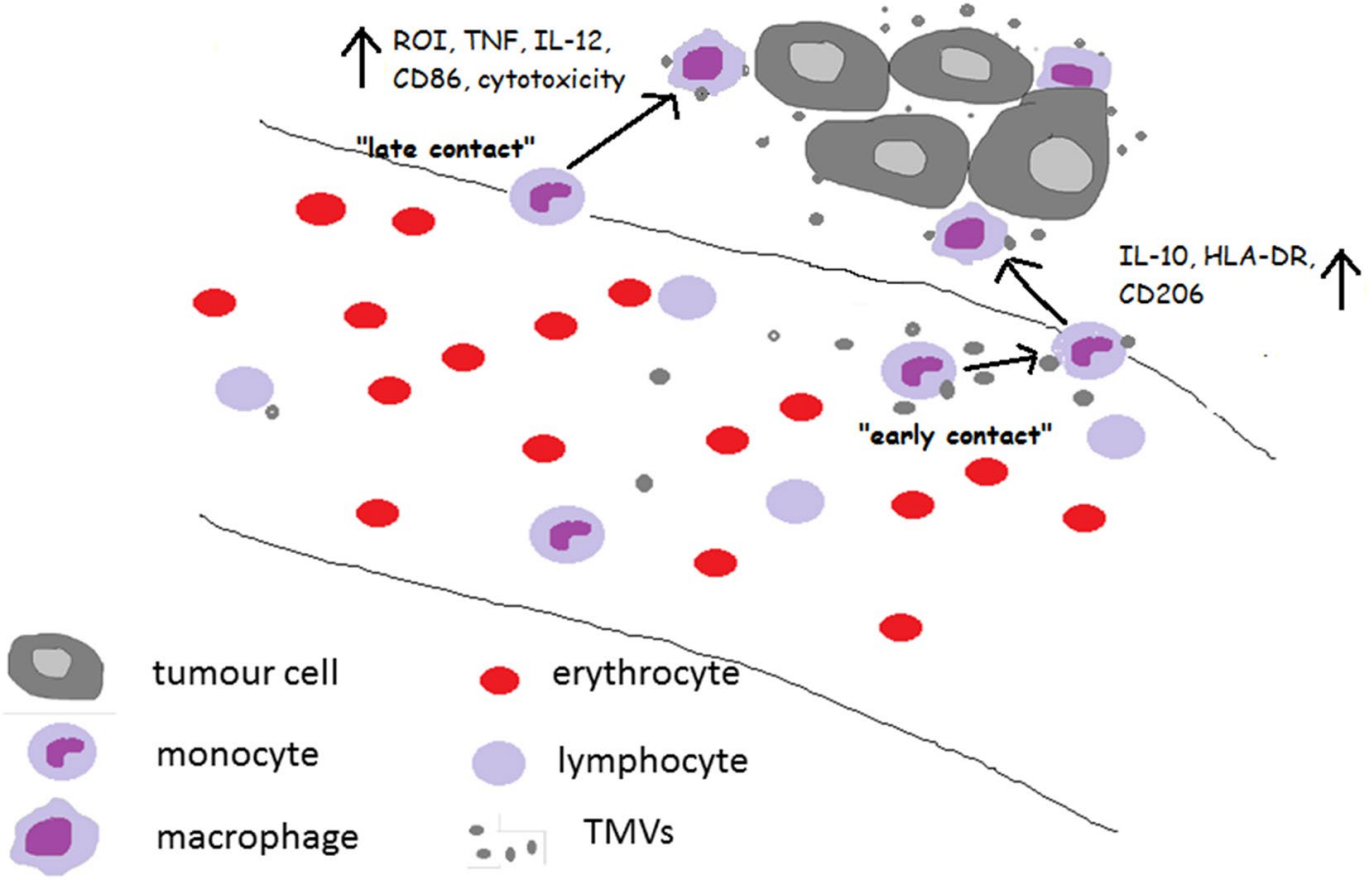
Presentation of the hypothetical interactions of monocytes/macrophages with TMVs in the blood and in the tumour bed. Early contact of monocytes with TMVs resulting in M1/M2 mix polarization is schematically presented on the *right*. Late contact of macrophages with TMVs resulting in the induction of proinflammatory cells—*left side*. TMVs interactions with other immune cells as well as with tumour cells were omitted to simplify the scheme

The present data show that TMVs released by colon cancer cells influence differentiation of blood monocytes to macrophages, resulting in their mixed polarization status (M1/M2) (Fig. 10). Phenotype and biological properties of the latter depend on the time of the “first” contact of monocytes with TMVs, as well as on the TMVs origin, which is related to their cargo (proteins, lipids etc.). To compare the impact of TMVs on monocyte differentiation, four colon cancer cell lines with different growth potential were used. As a control, MVs from non-malignant HCV-29 cell line were implemented into the study. Also, in our model three different time regimens of TMVs exposition were used to mimic contact of monocytes with TMVs: i) in the peripheral blood (MDM + TMV0d), ii) in the peripheral blood and later during e.g. extravasation (MDM + TMV036d), iii) at the final stage of maturation (MDM + TMV6d) e.g. in the tumour bed.

It was previously reported that TMVs interact with human monocytes, leading to their activation [17]. The present study addressed the question whether TMVs may have an impact on monocyte differentiation and if so, what type of myeloid cells is generated as the result of this process. In the mouse model, tumour derived exosomes directed monocytes differentiation into myeloid-derived suppressor cells (MDSC), which were characterized by the expression of CD11b(+)Gr-1(+) markers [23]. Moreover, Valenti et al. showed impaired differentiation of monocytes to dendritic cells in the presence of tumour exosomes and cytokines (GM-CSF, IL-4) [24]. This study shows that TMVs alone may impact differentiation of monocytes to macrophages, however, it should be kept in mind that we used a mixed population of vesicles, not just limited to exosomes. Depending on the time of the “first” contact between monocytes/MDM and TMVs (but not “normal” MV), differences in MDM morphology, phenotype, cytokine secretion, ROI production and cytotoxicity against tumour cells in vitro were observed. We observed that TMVs present in the MDM culture induced transient cell cluster formation. The mechanism of this phenomenon is not clear, however, we suggest that this is related to hyaluronan, which is present in TMVs and MVs [25]. MDM differentiated in the presence of TMVs are bigger and more granular than control MDM. All of them showed intracellular expression of CD68 and surface expression of CD14, however, the highest CD14 expression (marker of M1 cells [26]) was observed on MDM + TMV6d.

Expression of CD206 was significantly higher on MDM + TMV0d and MDM + TMV036d than on control MDM and MDM + TMV6d. Lower expression of HLA-DR on MDM + TMV 6d was observed, but it was not significant. CD80 expression was elevated by all TMVs except for TMV_SW620_ (highly metastatic cell line). Expression of CD86 was significantly higher on MDM + TMV 6d. TMV_SW480_ decreased expression of CD163 on MDM. No differences in CD115 (M-CSF R) expression were observed after contact with TMVs. We should stress out that these observation are specific for TMVs and not observed after contact with control MVs used in our study.

The phenotype of MDM + TMV6d (low HLA-DR, CD206, high CD86) combined with morphological features may indicate TAM with mixed polarization. Simultaneous phosphorylation of STAT1 and STAT3, combined with the expression of miR-155 and miR-9 supports this hypothesis. The distinct expression of HLA-DR on TAM was also observed by others [27] and usually it was attributed to hypoxia conditions in the tumour bed [28]. Based on our findings, we suggest, that the HLA-DR expression may be also influenced by the previous stimulation/contact with TMVs. In parallel, elevated expression of CD86, correlates with clinical data that most macrophages distributed along the invasive margin of colorectal carcinoma are CD86^+^ [29, 30]. Heterogeneity of TAM phenotype was described in different tumour types [31] and at different locations of the same tumour [32, 33] e.g. in colon cancer patients, CD80^+^, CD86^+^ and HLA-DR^+^ [34], as well as CD163^+^, CD86^+^, CCR2^+^ cells were described [7].

The elevated expression of CD206 (M2 marker) on MDM + TMV0d and MDM + TMV036d may indicate M2 polarization. Control MDM were grown in culture medium without growth factors to develop M0 phenotype [21], however, the elevated expression of CD206 may indicate spontaneous predominance of M2 over M1 phenotype in these cells [35]. The upregulation of the mannose receptor in tumour macrophages was accompanied by increase of miR-511-3p [36], which corroborates our data (Fig. 5e). Moreover, we observed dynamic increase of CD206 expression at day 1 of culture, which correlated with dynamic changes in different miR expression during the first day of differentiation [18].

Low expression of CD115 (M-CSFR) is rather a sign of non-M2 cells, as M-CSF directed differentiation towards M2 [37]. The decrease of CD115 on macrophages was previously observed by Rovida et al. and explained by its shedding by proteases from macrophages undergoing activation [38].

Different phenotype of macrophages infiltrating tumour site may allow to predict their activation status, which subsequently may anticipate their response to tumour. In fact, TAM phenotyping may be more informative than density/number of infiltrating cells for patient prognosis [33, 37].

In this study the activation of MDM was measured by cytokine secretion, ROI production and cytotoxicity against tumour cells. Cytokine secretion by MDM and their phenotype, depended on time and frequency of the contact with TMVs and TMVs origin. The strongest secretion of TNF and IL-12 was observed when TMVs were added at the final stage of differentiation (MDM + TMV6d). In comparison, MDM + TMV0d released significantly more IL-10 than other types of MDM. MDM differentiated in the presence of TMV_SW480_ were the weakest producers of IL-10, while MDM + TMV_LoVo_6d and MDM + TMV_SW480_6d, produced the highest amounts of TNF, which may be related to their growth potential. It is of note that MDM differentiated after a prolonged contact with TMVs (MDM + TMV036d) secreted the lowest amounts of TNF and IL-12. This is in keeping with previously described deactivation of monocytes/macrophages by tumour cells [22], which may be observed also after TMVs contact, presumably via their hyaluronan component [39].

The most potent ROI producers were MDM + TMV6d, but it seemed to be TMVs-origin dependent as TMV_Caco2_ did not induce production of ROI. MDM + TMV6d were more cytotoxic/cytostatic to tumour cells, most likely due to the cytotoxicity mediated by ROI and proinflammatory cytokines. Our data corroborates with the observation that TAM isolated from spheroids of human colorectal cancer expressed antitumour potential probably via secretion of proinflammatory mediators [9].

The observed differences between MDM + TMV0d and MDM + TMV6d (early and late contact) may be due to the regulation of the differentiation process via e.g. microRNA. The first scenario is, that the fate of microRNA in MDM is influenced not only by cytokines and nearby cells [18, 40] but also by TMVs present in the local environment. In keeping with this, we observed different miR expression profile in MDM + TMV0d and MDM + TMV6d. MDM after early contact with TMVs had upregulated miRs expression characteristic for M2-like cells (miR-21,-511,-9). The highest expression of these miRs was observed in the case of TMV_Caco2_, which induced differentiation of MDM with a lower proinflammatory capacity. MDM + TMV6d expressed higher levels of miR-155 in comparison to MDM + TMV0d and MDM control for all tested TMVs. MDM + TMV_LoVo_6d and MDM + TMV_SW480_6d expressed also more miR-378. Taken together, the miR profile may suggest predominance of M2 cells after early contact and more proinflamatory cells after late contact with TMVs. Morphology of MDM + TMV6d (“fried egg”-shaped cells), and the cytokine secretion support the hypothesis of switch (at least in part of the cells) towards proinflammatory activity.

The second possibility, which complements the first one, is that miRNAs are carried by TMVs themselves. MVs were described before as safe transporters for miRNAs involved in regulation of cellular differentiation [41, 42]. TMVs used in our experiments carried miR-106a, -146a, -155, -222, -223, -378 which are crucial for macrophage differentiation and activation processes [43, 44], however, quantitative assessment of this phenomenon requires further confirmation. The direct transfer of miR cannot be excluded, except for miR-511 which was not detected in TMVs.

Our and others’ [45] results support the observation that TAM may have distinct properties, which do not fit perfectly the classical M1/M2 definition. We conclude that MDM differentiated in the presence of TMVs represent a mixed phenotype (M1/M2). We suggest that late contact with TMVs predisposes MDM to more proinflammatory activity. Hypothetical scenario is presented in Fig. 10. Furthermore, we suggest that biological activity of MDM is more important than their number and phenotype during tumour progression. Although, we do not have a formal proof, we suspect that removal of TMVs from blood in order to delay the first contact with monocytes, may improve macrophage activity against cancer. The idea is not new as Ichim et al. proposed a physical approach in order to remove tumour exosomes from the body fluids of the cancer patients [46]. Also, the isolation of monocytes followed by their differentiation ex vivo and “education” with TMVs may increase their anti-tumour potential. Again, the idea was already implemented, but with dendritic cells [47].

Taken together, MDM differentiated in vitro with TMVs fit the “colour wheel” classification of macrophage polarization as different “shades” of green [48], which we think, may be induced by TMVs.

## Conclusions

TMVs modulated activity of monocytes and their differentiation towards macrophages. The final polarization status of macrophages was dependent on TMVs origin and the time of contact with them. We suggest, that in the case of colon cancer, late contact of MDM with TMVs induced their mix polarization with significant proinflammatory potential.

## Abbreviations

TMVs: tumour-derived microvesicles
EVs: extracellular vesicles
FBS: foetal bovine serum
MDM: monocyte-derived macrophages
MFI: mean fluorescence intensity
TNF: tumour necrosis factor
IL: interleukin
TGFβ: transforming growth factor beta
PMA: phorbol 12-myristate 13-acetate
ROI: reactive oxygen intermediates
MDSC: myeloid-derived suppressor cells
TAM: tumour associated macrophages
RQ: relative quantity
GM-CSF: granulocyte-macrophage colony-stimulating factor
M-CSF: macrophage colony-stimulating factor
miR: microRNA
